# Adaptation and validation of the Charlson Index for Read/OXMIS coded databases

**DOI:** 10.1186/1471-2296-11-1

**Published:** 2010-01-05

**Authors:** Nada F Khan, Rafael Perera, Stephen Harper, Peter W Rose

**Affiliations:** 1 Department of Primary Health Care, University of Oxford, Old Road Campus, Oxford, OX3 7LF, UK; 2 Mill Stream Surgery, Benson, Wallingford, OX10 6RL, UK

## Abstract

**Background:**

The Charlson comorbidity index is widely used in ICD-9 administrative data, however, there is no translation for Read/OXMIS coded data despite increasing use of the General Practice Research Database (GPRD). Our main objective was to translate the Charlson index for use with Read/OXMIS coded data such as the GPRD and test its association with mortality. We also aimed to provide a version of the comorbidity index for other researchers using similar datasets.

**Methods:**

Two clinicians translated the Charlson index into Read/OXMIS codes. We tested the association between comorbidity score and increased mortality in 146 441 patients from the GPRD using proportional hazards models.

**Results:**

This Read/OXMIS translation of the Charlson index contains 3156 codes. Our validation showed a strong positive association between Charlson score and age. Cox proportional models show a positive increasing association with mortality and Charlson score. The discrimination of the logistic regression model for mortality was good (AUC = 0.853).

**Conclusion:**

We have translated a commonly used comorbidity index into Read/OXMIS for use in UK primary care databases. The translated index showed a good discrimination in our study population. This is the first study to develop a co-morbidity index for use with the Read/OXMIS coding system and the GPRD. A copy of the co-morbidity index is provided for other researchers using similar databases.

## Background

Studies of patient health should take into consideration any independent predictors that will affect the outcome of interest. Individual disease status is an important predictor of mortality and health care usage especially in studies of older patients, and in many cases, subjects may have more than one co-existing illness at the same time. Investigators may wish to conduct risk adjustment for the additional health effects of these co-morbid diseases.

Previous research has led to the development of summary comorbidity measures which classify patients according to their disease burden [1-4]. The most widely used and validated index of comorbidity was developed by Charlson and colleagues in the late 1980s [5,6]. The Charlson index includes 17 categories of comorbid disease weighted based on their association with 1 year all-cause mortality. Because the Charlson index is weighted and allows for additive scoring, it can take into account both the number and the severity of comorbidity to provide a summary of disease burden for each individual patient [6]. The index has been validated in several different populations, and has been widely used in studies involving cancer patients and survivors [7-11].

Recognizing the potential for its use in large database studies that require risk adjustment for individual patients, the Charlson index has previously been adapted for use with administrative data [12-15]. These adaptations involve searching individual level hospital claims data for codes corresponding to the Charlson index categories. However, these adaptations generally apply only to ICD-9-CM coded data, an international coding system for classification of diseases, symptoms and signs. There is no current translation of the Charlson index for Read and OXMIS coded data, two systems which are based on ICD-9-CM and are widely used in British primary care. Data using the Read and OXMIS coding system has recently been made more readily available from the General Practice Research Database (GPRD), a UK-based database of clinical primary care records. The GPRD is the world's largest source of anonymised longitudinal data from primary care, and currently contains information on 3.6 million active patients from 450 general practices in the UK [16]. With increasing use of the GPRD for academic and epidemiological research, there is a need for a GPRD-compatible research tool that will allow categorization and adjustment for patient comorbidity.

The main aim of this paper is to develop a comorbidity index based on the Charlson index for use with Read/OXMIS coded data. We also describe the performance of the new measure by testing whether comorbidity is associated with increased mortality in a cohort of patients from the GPRD. It is our hope that the newly developed and tested translation of the Charlson score can be used by other researchers working with Read/OXMIS coded data and the GPRD.

## Methods

### Development of Read/OXMIS codes lists

**Figure 1:**
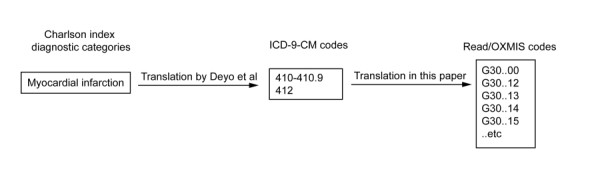
**Translation of Charlson index to Read/OXMIS codes**.

The original Charlson index consists of 17 diagnostic categories which provide the basis for assigning weighted scores to each comorbid disease. Deyo et al [12] describe a validated translation of each diagnostic category of the Charlson index to ICD-9-CM codes. We used the ICD-9-CM codes suggested by Deyo et al to guide development of the Read/OXMIS code lists used in this comorbidity index. Figure 1 summarizes our process for translation of the index to Read/OXMIS codes using one of the Charlson diagnostic categories, myocardial infarction, as an example.

Using definitions provided by Deyo et al for each Charlson diagnostic category, we searched the General Practice Research Database Medical Dictionary (Version 0.3.7, Copyright © 2004) for potentially relevant Read/OXMIS codes. This dictionary includes the GPRD medical code for the type of event, the Read/OXMIS code for the event and a description of the medical term. We identified potential Read/OXMIS terms using two search strategies. Firstly, we used specific terms in the ICD-9-CM description of the event to search the GPRD dictionary. Read codes have a hierarchical structure, with a top level code for a disease category branching into more precise and specific codes. Therefore, our second strategy involved identifying relevant top level Read codes and including all lower level codes. By using the wild card (*), and hierarchies of Read codes, we generated a list of all potentially related codes. We conducted these Read/OXMIS searches for 16 of the diagnostic categories used in the Charlson index. We treated the cancer codes separately, and included all Read codes starting with 'B', but excluding all codes for benign cancer (B7), cancer in situ (B8) and neoplasms of uncertain behaviour (B9). We tried to be over-inclusive in the searching and used broad search terms when possible.

Two clinicians experienced in the use of Read codes (PWR and SH) independently reviewed the list of all Read/OXMIS codes identified through searches of the GPRD medical dictionary. The clinicians selected relevant Read/OXMIS codes and rejected codes not corresponding to ICD-9-CM codes used in the Deyo adaptation of the Charlson index. A third clinician resolved any disagreement on coding. We calculated the degree of inter-rater agreement between the two clinicians reviewing Read/OXMIS code lists using the kappa score, which provides an estimate of the level of agreement between the two raters above that occurring due to chance. The final list of Read/OXMIS codes in this adaptation of the Charlson index is available in Additional file 1.

13 Read/OXMIS codes were used in more than one diagnostic category. These overlaps only occurred between diabetes and peripheral vascular disease (i.e. 'gangrene diabetic' was coded as both 'diabetes' and 'peripheral vascular disease'), and between diabetes and diabetes with complications. One clinician (PWR) determined that the codes should be classified as diabetes codes.

### Data source

The GPRD is the world's largest anonymised database of primary care records [16]. Practices participating in the GPRD record data on clinical diagnoses, test results, prescriptions and referral data from primary care. Clinical data is coded using Read/OXMIS codes, along with the date of original onset for chronic or recurrent conditions. GPRD Recording Guidelines direct practices to provide a record of all significant morbidity events in the patient's medical history, including a summary of events that occurred before the patient joined the practice [17]. The data from practices undergoes quality control procedures and several validation studies have shown a high level of data completeness within the GPRD [18].

### Validation dataset

As part of a study looking at the unmet needs of long-term survivors of cancer, we received a dataset containing primary care records between 01/01/1987 and 30/06/2006 for all patients in the GPRD with a diagnosis of breast, colorectal or prostate cancer and more than five years survival. We also received data on a control population of patients with no record of breast, colorectal or prostate cancer, matched to the cancer survivors by age, gender and practice on a ratio of 1:4. The dataset included data on 18707 breast cancer survivors, 5773 colorectal cancer survivors, 4856 prostate cancer survivors, and 117105 control patients (total *n *= 146 441 patients). We used individual level clinical data in this dataset to test the adapted Charlson index.

### Assessing the comorbidity measure

Following translation of the Charlson index to Read/OXMIS codes, we tested whether an increasing comorbidity score was associated with increased patient mortality. To achieve this, we applied the adapted weighted Charlson index to the patient cohort obtained from the GPRD. Our adapted comorbidity score used the original Charlson score which does not include age, however, Charlson and colleagues have also developed a combined age-comorbidity index [19].

Cox proportional hazards models were fit with mortality from July 1 2001 to 31 August 2006 as the dependent variable. Charlson score was coded as a continuous ordinal indicator variable, and was included along with age as explanatory variables. Survival was measured in days and associations are reported using hazard ratios with Charlson score of 0 as the referent group. All analyses were conducted using Stata MP (Version 10, College Station, TX).

### Discriminatory power of the model

The area under a receiver operating characteristic (ROC) curve, or c-statistic, can be used to quantify how well a predictor based on a number of variables discriminates a dichotomous outcome [20]. We used a logistic model to estimate the relationship between death (dichotomous outcome coded 0/1) and the Charlson index, after adjusting for age, quintiles of the the Index of Multiple Deprivation (IMD) and gender, before producing the ROC curve. Model discrimination was assessed by the area under the ROC curve.

### Adjusting variables

We included age in 2001, gender and socioeconomic status in the models for adjustment. Age was categorized in 5 groups of similar sizes (30-49, 50-59, 60-69, 70-79, 80+). The GPRD dataset includes an Index of Multiple deprivation (IMD) score to estimate socioeconomic status at practice level. The IMD covers a range of indicators including income, employment, health deprivation and disability, education, skills and training, housing, and geographical access to services for each small area in the UK [21]. IMD scores were grouped into quintiles based on the spread of scores within each country in the UK.

## Results

### Coding exercise

The inter-rater agreement between the two clinicians for including Read/OXMIS codes in the Charlson index was 84.6%, with a kappa of 0.45, indicating a moderate level of agreement [22]. Including the cancer codes, a total of 3156 Read/OXMIS codes were included in this adaptation of the Charlson index.

### Characteristics of the cohort

The mean age of the cohort was 73.7 (SD 12.5), and 73.5% of the patients were female. The high percentage of female patients is due to the high proportion of breast cancer survivors and gender matched controls in the cohort. Table 1 shows the frequency and percentages of patients with each of the diagnoses included in the comorbidity index.

**Table 1:** Frequency of comorbid disease in validation cohort

Charlson Diagnostic category	**Weighted score in Charlson index **[6]	Number of patients in our dataset
AIDS	6	8 (0%)
Cerebrovascular disease	1	9,028 (4.4%)
Chronic pulmonary disease	1	27,698 (13.5%)
Congestive heart disease	1	9,217 (4.5%)
Dementia	1	3,624 (1.8%)
Diabetes	1	14,418 (7.0%)
Diabetes with complications	2	2,544 (1.2%)
Hemiplegia and paraplegia	2	505 (0.25%)
Mild liver disease	1	416 (0.2%)
Moderate or severe liver disease	3	96 (0.05%)
Myocardial infarction	1	6,512 (3.2%)
Peptic ulcer disease	1	6,943 (3.4%)
Peripheral vascular disease	1	6,731 (3.3%)
Renal disease	2	12,624 (6.1%)
Rheumatological disease	1	8,140 (3.9%)
Cancer	2	34,750 (16.9%)
Metastatic tumour	6	2,116 (1.0%)
No comorbid disease	-	60,585 (29.4%)

**Total**		205,955

* Patients can be counted more than twice if they have more than one comorbid disease, therefore the total will not equal 146 441 (the number of patients in the cohort)

**Figure 2:**
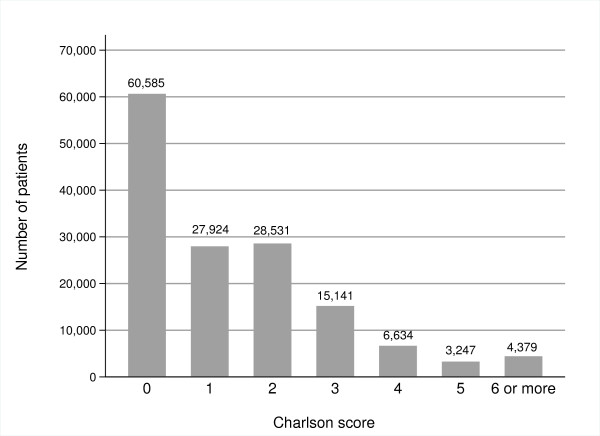
**Breakdown of Charlson score in patient dataset**.

The original Charlson index weights each disease category on the strength of its association with mortality. Using the original Charlson weights for each disease category, the breakdown by index score in our dataset is shown in Figure 2.

Most of the patients in the validation dataset had no comorbid disease (n = 60585). There were a few patients with a very high Charlson score above 5. There was a strong positive association between increasing Charlson score and increasing age (p < 0.0001).

### Patient mortality

**Figure 3:**
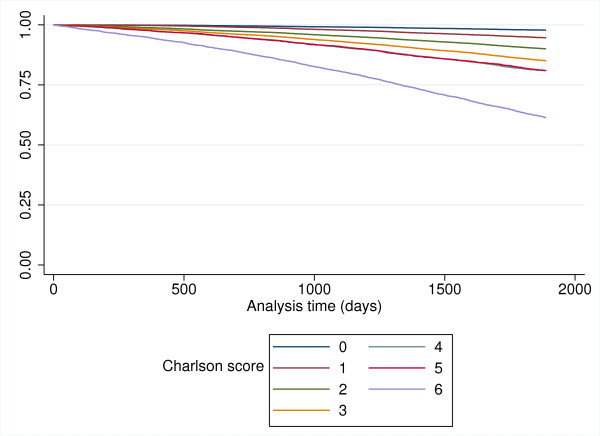
**5 year mortality stratified by Charlson score**.

In total, 11,490 patients died during the five-year period from July 1 2001 to August 31 2006. Figure 3 shows the survival curves for the population stratified according to Charlson score.

Mortality was significantly associated with a Charlson score of 1 or more, with a positive increasing association as Charlson score increases (Table 2). There was an increased risk of death amongst older patients and amongst males.

**Table 2:** Adjusted risk of death and 95% CI for 5 year mortality

	Adjusted Hazard ratio*	95% Confidence Interval
Charlson score		

0	1	

1	1.87^†^	1.74 - 2.02

2	3.68^†^	3.45 - 3.93

3	4.71^†^	4.40 - 5.05

4	5.47^†^	5.06 - 5.91

5	5.25^†^	4.77 - 5.78

6 or more	14.21^†^	13.21 - 15.28

		

		

30-49	1	

50-59	1.07	0.885 - 1.29

60-69	1.39^†^	1.16 - 1.66

70-79	2.98^†^	2.51 - 3.53

80+	10.51^†^	8.89 - 12.43

		

IMD quintile^‡^		

0*	1	

1	1.00	0.94 - 1.06

2	1.04	0.98 - 1.09

3	1.05	1.00 - 1.12

4	0.97	0.91 - 1.03

		

Gender		

Female	1	

Male	1.16^†^	1.12 - 1.21


* Adjusted for age, gender, and quintile of IMD

† Significant at the p < 0.0001 level

‡ A low rank indicates the least deprived, and a high rank indicates the most deprived

### Discrimination of the model

**Figure 4:**
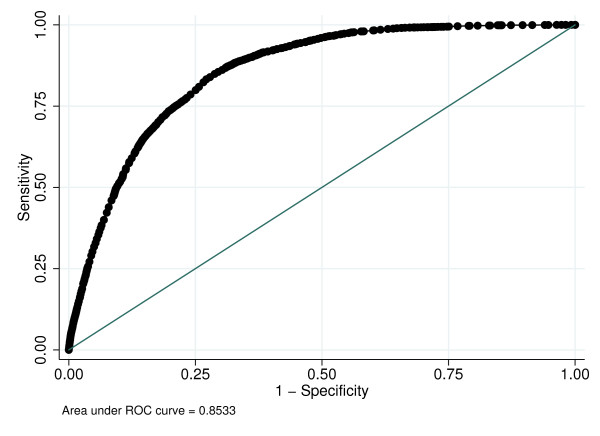
**ROC curve for logistic regression model**.

The discrimination of the logistic regression model was good, with an area under the curve (AUC) of 0.853 (Figure 4). This indicates that the adaptation of the Charlson index is a good predictor of mortality in the validation dataset.

## Discussion

We have translated a commonly used comorbidity index into Read/OXMIS codes for use with UK primary care databases. In a cohort of cancer survivors and matched controls, a higher comorbidity score in this adaption of the Charlson index was associated with an increased risk of mortality after adjusting for age, deprivation scores and gender. The translated comorbidity index showed a good discrimination our study population. To our knowledge, this is the first study to develop a comorbidity index for use with this disease coding system.

Although this adaptation of the Charlson index can be applied to any Read/OXMIS coded dataset, we hope that our adapted version of the Charlson index will be especially useful to the increasing number of researchers conducting work using the GPRD. GPRD data provides an opportunity to conduct large scale epidemiological research in primary health care use, health outcomes and pharmacology. The Medicines and Healthcare products Regulatory Agency (MRHA), which manages the GPRD, has recently announced plans to link GPRD data with Hospital Episodes Statistics (HES), cancer registrations and Office for National Statistics databases. These linkages will increase the value of conducting research using the GPRD, as researchers will be able to trace patient pathways through primary and secondary care. Our adaptation of the Charlson index is available in Additional file 1, and can be imported into statistical software for use with other datasets. This index can be used to quickly categorize patients into different comorbidity levels, and will add explanatory power when conducting analyses using Read/OXMIS coded datasets and the GPRD.

We tested the association between increasing Charlson score and mortality, which was the primary outcome used in the development of the original score. Our results confirm the hypothesis that patients with a greater burden of disease will die sooner. One unexpected result was a large jump in the risk of mortality in patients with a Charlson score of 6 or above. This is likely due to the number of patients in our cohort with metastatic cancer, which generally has a very poor prognosis in the small but high risk group of patients with a score of 6 or more [23].

### Limitations

This comorbidity index performed well in our validation exercise, however, there are several areas where the model may be inadequate. Firstly, it is possible that some codes were not included when developing the Read/OXMIS code lists for assessment. By using broad search terms, hierarchical searches of Read codes, and two reviewers to independently assess records, fewer potentially relevant Read/OXMIS codes were excluded from the final adaptation of the Charlson index. Secondly, we used the original disease weights developed by the authors of the original Charlson index almost twenty years ago. One recent criticism of the Charlson index is that certain diseases have an improved prognosis since the original score was developed. For instance, according to the original Charlson weighting, a positive AIDS disease status carries an equivalent mortality risk to a diagnosis of metastatic cancer. Only eight patients in our dataset were diagnosed with AIDS, therefore, this issue is unlikely to affect our validation results. In studies where a larger proportion of individuals are HIV/AIDS positive, investigators may wish to use updated weights for HIV/AIDS taking into account that the burden of disease and mortality is lower now than in the 1980s [24].

Thirdly, recording of clinical outcomes in primary care settings may be incomplete; a recent study demonstrated that even major outcomes such as cancer may not be recorded in patient electronic records [25]. Although the GPRD data is subject to a number of quality checks, it is possible that disease recording is incomplete. However, many of the previous adaptations of the Charlson index have used administrative data, where patient history and comorbid disease may not be as recorded as accurately as the clinical data available in datasets such as the GPRD [26,27]. Omissions of major comorbid diseases can result in an incorrect final Charlson score in any study. These omissions are not an intrinsic limitation in the tool that we have developed, but may affect its functional ability in datasets such as the GPRD. Future work should continue to validate the accuracy of disease coding in administrative datasets and the GPRD.

Our validation population of long-term cancer survivors is unusual; these patients are older and sicker than the general population. The cancer survivors have a Charlson score of at least 2 and a high proportion are female owing to the high number of breast cancer survivors. Other patient cohorts using this adapted comorbidity index will likely have different trends in mortality and consultation behaviour. Future studies should apply this Charlson adaptation to other study populations to measure mortality and use of primary care services. We were also unable to consider race or ethnicity in the analysis as this information is not routinely collected in the GPRD. These limitations, however, do not affect the development and translation of the Charlson index to Read/OXMIS codes, but may affect the results of the validation exercise.

## Conclusions

In conclusion, we have developed an adaptation of the Charlson comorbidity index for use in Read/OXMIS databases and the GPRD which predicts 5-year mortality in a cohort of patients. Our adaptation is provided in a downloadable format (Additional file 1) for other researchers using similar databases. With increasing use of large datasets for epidemiological research, researchers must consider how disease status will affect their outcomes of interest. Tools such as the Charlson index can provide a summary of comorbidity for use in large studies, and this paper demonstrates the utility of an adaptation of the Charlson score in primary care coded data.

## Abbreviations

ICD-9: International Classification of Diseases; IMD: Index of Multiple Deprivation; GPRD: General Practice Research Database; ROC: Receiver operating characteristic

## Competing interests

The authors declare that they have no competing interests.

## Authors' contributions

NFK conceived the study, carried out all analyses and drafted the manuscript. PWR participated in the design and coordination of the study, and along with SH reviewed all codes. RP provided expert statistical advice. All authors read and approved the final manuscript.

## Pre-publication history

The pre-publication history for this paper can be accessed here:

http://www.biomedcentral.com/1471-2296/11/1/prepub

## Supplementary Material

Additional file 1 **Adapted Charlson score**. This file contains the Read/OXMIS codes relating to this adaptation of the Charlson index. Click here for file
